# Inheritance analysis and mapping of quantitative trait loci (QTL) controlling individual anthocyanin compounds in purple barley (*Hordeum vulgare* L.) grains

**DOI:** 10.1371/journal.pone.0183704

**Published:** 2017-08-23

**Authors:** Xiao-Wei Zhang, Qian-Tao Jiang, Yu-Ming Wei, Chunji Liu

**Affiliations:** 1 CSIRO Agriculture & Food, St Lucia, Queensland, Australia; 2 Triticeae Research Institute, Sichuan Agricultural University, Wenjiang, Chengdu, China; University of Tasmania, AUSTRALIA

## Abstract

Anthocyanin-rich barley can have great potential in promoting human health and in developing nutraceuticals and functional foods. As different anthocyanin compounds have different antioxidant activities, breeding cultivars with pre-designed anthocyanin compositions could be highly desirable. Working toward this possibility, we assessed and reported for the first time the genetic control of individual anthocyanin compounds in barley. Of the ten anthocyanins assessed, two, peonidin-3-glucoside (P3G) and cyanidin-3-glucoside (C3G), were major components in the purple pericarp barley genotype RUSSIA68. Quantitative trait locus (QTL) mapping showed that both anthocyanin compounds were the interactive products of two loci, one located on chromosome arm 2HL and the other on 7HS. However, the two different anthocyanin components seem to be controlled by different interactions between the two loci. The effects of the 7HS locus on P3G and C3G were difficult to detect without removing the effect of the 2HL locus. At least one copy of the 2HL alleles from the purple pericarp parent was required for the synthesis of P3G. This does not seem to be the case for the production of C3G which was produced in each of all the different allele combinations between the two loci. Typical maternal effect was also observed in the inheritance of purple pericarp grains in barley. The varied values of different compounds, coupled with their different genetic controls, highlight the need for targeting individual anthocyanins in crop breeding and food processing.

## Introduction

Anthocyanins are a group of water-soluble flavonoids responsible for the attractive colours of most fruits, vegetables and cereal grains [1]. They have been recognised as having the ability to scavenge free radicals, which cause oxidative stress in human cells [2, 3], and can potentially offer various health benefits and play important roles in protection against oxidative damage and detoxification, as well as in processes related to the immune system. For example, it has been reported that anthocyanins have the effects of anti-inflammatory [4–7], anti-obesity [8], anti-cancer [9] and hypoglycaemic effects [10, 11]. These bio-functionalities render anthocyanins considerable potential in the food industry as safe and effective food colourants [12], nutraceuticals [13] and functional foods [14].

Anthocyanins in cereals have gained increasing attention because they are commonly consumed foods [1]. Pigmented grains rich in anthocyanins have been identified as promising ingredients for the development of cereal-based functional foods as they are a source of natural antioxidants [15–18].

Barley is widely adapted to diverse environments and it is the fourth most abundant cereal in both acreage and tonnage harvested (http://faostat.fao.org). Most of the world production of barley is used for animal feeds or malted for use in alcoholic and non-alcoholic beverages. However, barley is still a major food source in poorer countries [19]. In more developed societies it has recently been classified as a true functional food ingredient [20, 21]. Previous studies have shown that pigmented barley can have great potential in promoting human health and in the development of nutraceuticals and functional foods [22]. A study on barley found that the antioxidant and anti-mutagenic effects of anthocyanins were not only identified in food products but also from fermented broth [23].

In-vitro assays showed that anthocyanin compounds varying in sugar linkages possess different antioxidant capacities [24, 25]. Hence, characterization of the composition of anthocyanin compounds in barley cultivars is an important consideration in the selection of anthocyanin-rich barley breeding lines [1, 26]. Previous studies have shown significant variation in not only total anthocyanin content (TAC) but also in anthocyanin compositions among various barley genotypes [27].

There are several reports on the genetics of anthocyanin pigmentation in grain [28–30] and several other tissues [31, 32] in barley. However, these studies were all based on segregation of overall colour of the tissue in concern. Considering that different anthocyanin compounds may have different values [24, 33, 34] and that grains with similar colour can have highly different anthocyanin compositions [26], we investigated the genetics of individual anthocyanin compounds in barley and the results were presented in this publication.

## Materials and methods

### Plant materials

Six populations between three purple pericarp barley accession and two non-pigmented commercial cultivars were developed and used in this study. They included:

1. RUSSIA68 / Gairdner F4 (POP1): 132 lines;
2. Baudin//RUSSIA68/Gairdner, 3-way cross F3 (POP2): 112 lines;
3. ETH002/Gairdner F2 (POP3): 200 lines;
4. Gairdner/ETH002 F2 (POP4): 150 lines;
5. RUSSIAN68/Gairdner F2 (POP5): 170 lines, and
6. Gairdner/RUSSIAN68 F2 (POP6): 190 lines.

RUSSIA68, RUSSIAN68 and ETH002 are purple pericarp and hulless barley accessions. ETH002 was originated from Ethiopia and the other two from the former Soviet Union. All the populations were generated in a glasshouse at the Queensland Bioscience Precinct in Brisbane, Australia. The first population was used for QTL mapping and the second one was used for assessing the effects of the QTL identified from the mapping population. The other four populations were used to assess the maternal effect observed from the first two populations.

### Evaluation of anthocyanins in barley grains

Harvested grains were dried in a ventilated oven at 28°C for 2 weeks and stored in an air-dried state at room temperature (20°C) until analysed. Anthocyanins in grains were extracted according to the methods described by Abdel-Aal and Hucl [35] with slight modification. Grains were ground using a ball mill (Retsch, Haan, Germany) then sifted with a 0.2 mm screen. Acidified methanol (Methanol: 1.0 N HCl = 85:15) was added to flour at the ratio of 10:1 (volume: weight) and then the pH of the mixture was adjusted to 1.0. The mixture was shaken on a vibrax set to 250 rpm at 4°C for 24 hours and then centrifuged at 10,000 × g for 25 min. The supernatant was pipetted into a clean tube and analysed with Liquid Chromatography Multiple Reaction Monitoring Mass Spectroscopy (LC-MRM-MS). For each line, the anthocyanin extraction was performed in triplicate with different grains and the anthocyanin content was calculated based on the average of the three replicates. A *t* test was used for statistical analyses.

Based on previous studies on anthocyanins in pigmented barley [1, 27], ten anthocyanin compounds (S1 Table) were initially selected for analysis. A volume of 10 μL sample or standard was injected to a Shimadzu Nexera UHPLC, passed through a Kinetex C18 1.7 μm column (Phenomenex 2.1 mm × 100 mm) at 0.4 mL/min over 15 minutes at 60°C. The mobile phase consisted of solvent A (formic acid: water = 0.1: 99.9) and solvent B (formic acid: acetonitrile: water = 0.1: 90: 9.9). The compounds were eluted with the following gradient: a linear gradient from 5% to 45% solvent B over ten minutes, followed by a linear gradient from 45% to 80% B over one minute, one minute at 80% B and an equilibration at 5% B. A 6500 QTRAP mass spectrometer (AB/Sciex) coupled to the HPLC was used to measure anthocyanin content. The MS parameters used were: curtain gas 35 psi, GS1 40 psi, GS2, 50 psi, ionspray voltage (IS) 5500 V and source temperature 500°C. The data were acquired by Analyst 1.6.2^™^ (AB/Sciex). Each anthocyanin was monitored by 8 precursor-to-product ion transitions and quantified using one transition. Peaks with a signal to noise ratio of > 7 were integrated using MultiQuant 3.0 (AB Sciex) and those matching corresponding standards were quantified.

### Molecular marker analysis

Leaves from each line of the POP1 and POP2 were collected and freeze-dried. Genomic DNA was extracted using the CTAB method as described by Anderson *et al*. [36]. SSR markers were selected according to their locations in published barley linkage maps [37, 38]. SSR markers were analysed based on the procedure described by Chen et al. [39] with some modifications. PCR reactions for the SSR markers were carried out in a total volume of 15 μL containing 50 ng genomic DNA, 0.2 μM of forward and reverse primer, 3 mM MgCl_2_, 0.2 mM dNTPs (including 600 nCi α-[33P] dCTP), and 0.3 U *Taq* DNA polymerase. PCR conditions used were obtained from the website of the GrainGenes (http://wheat.pw.usda.gov/GG3/). Mixtures of PCR products and loading dye were denatured at 95°C for 5 min and separated on denaturing 5% polyacrylamide gels. The gels were subsequently transferred onto filter papers and dried using a gel dryer before being exposed to Fuji X-ray film for 5–7days.

InDel markers were designed based on differences between the genomic sequences of *Hordeum vulgare* cv. *Morex* (http://plants.ensembl.org/Hordeum_vulgare/Info/Index) and that of a wild barley genotype AWCS079 obtained in our labs (unpublished). PCR reactions for InDel markers were performed in 25 μL mixtures containing 50 ng genomic DNA, 0.4 μM of forward and reverse primer, 3 mM MgCl_2_, 1 mM dNTPs, and 2.5 U *Taq* DNA polymerase. The PCR program included an initial denaturation at 94°C for 5 min, followed by 35 cycles of 94°C for 1 min, 60°C for 1 min, 72°C for 1 min, and a final extension at 72°C for 10 min. The PCR products were mixed with Gel-Red stain and separated on 2.5% agarose gels.

### Data analysis and QTL mapping

Statistical analyses were performed using the Minitab statistical software (version 17, Minitab, State College, PA, USA.). Linkage analysis was carried out using JointMap 4.0 [40] and map distances were estimated using the Kosambi equation [41]. QTL analysis and single marker analysis (SMA) were carried out using MapQTL 5.0 [42]. The Kruskal-Wallis test was used in a preliminary testing of associations between markers and anthocyanin content. For each trial, the LOD threshold (*p* < 0.01) was calculated based on 1,000 permutations test. According to the permutation test, a LOD threshold value was used to determine the presence of a QTL. The linkage map showing the QTL positions was drawn using Microsoft Excel.

## Results

### Inheritance of purple pericarp barley grains

**Fig 1:**
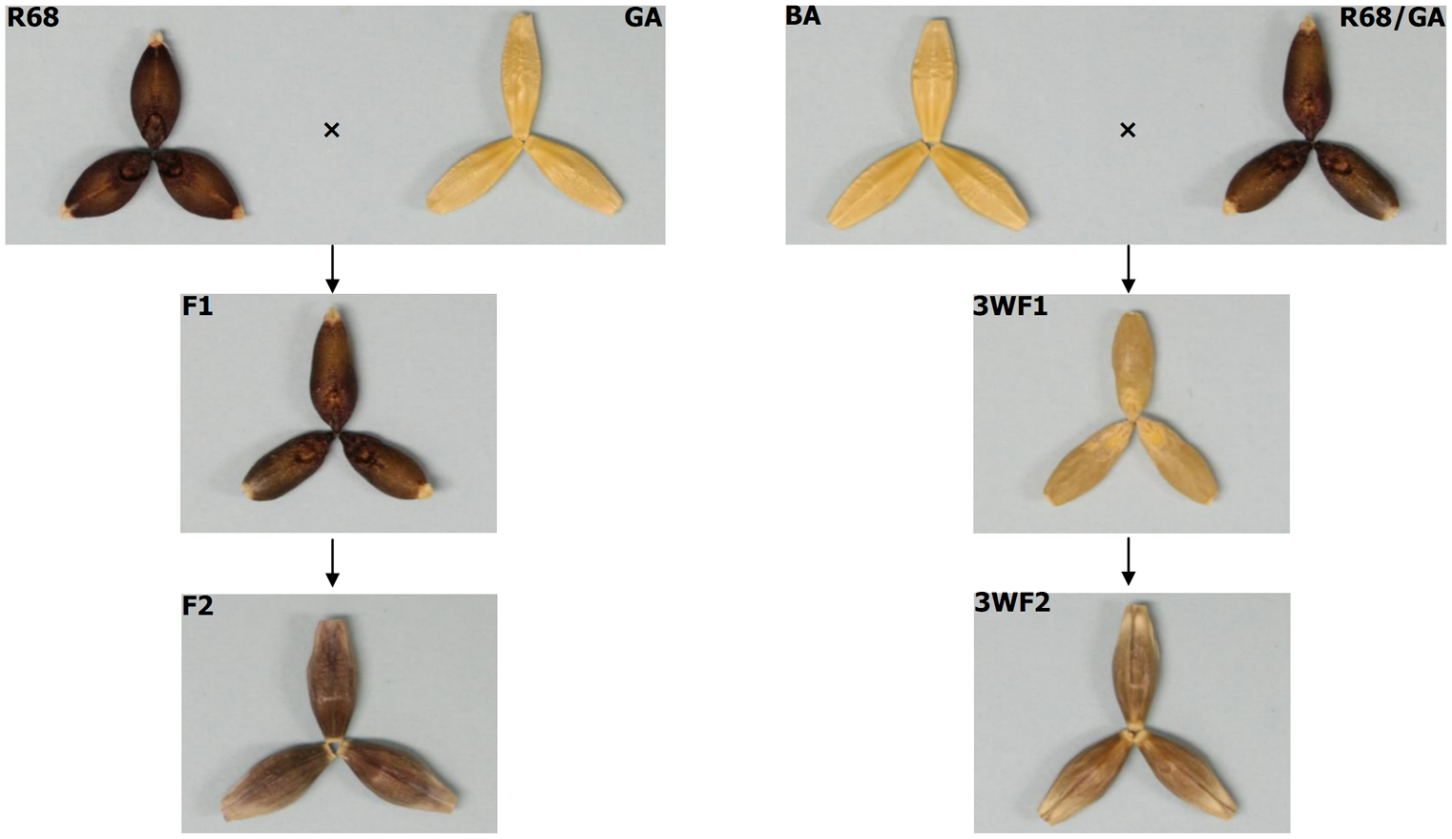
Showing the typical maternal effect in the inheritance of grain colour in purple barley. The parents, F1 and F2 grains of the mapping population (POP1) were showed on the left and those of the 3-way cross population on the right. R68 is the purple pericarp parent RUSSIA68 and GA the non-pigmented parent Gairdner. The colour intensities of the F1 or 3WF1 grains were similar to those of their respective female parents, those of the F2 grains were lighter than those of F1 and the grain of 3WF2 were darker than those of 3WF1.

It was noticed during the generation of both the mapping (POP1) and the validation (POP2) populations that the pericarp colour of F1 seeds was the same as the female parents and that the pericarp colour of F2 seeds was determined by the genotypes of F1 plants (Fig 1). Further, irrespective of their generations, all grains from a single plant had the same colour. These observations indicated typical maternal effect in the inheritance of purple pericarp colour. To verify this, four additional crosses (POP3, POP4, POP5 and POP6) between two different genotypes of purple pericarp barley (ETH002 and RUSSIAN68) and two non-pigmented commercial cultivars (Gairdner and Baudin) were generated. The maternal effect in the inheritance of pericarp colour was observed again in each of these crosses as that the colour of a grain is not determined by its own genotype but its mother’s.

### Characterization of anthocyanins in the mapping population

**Fig 2:**
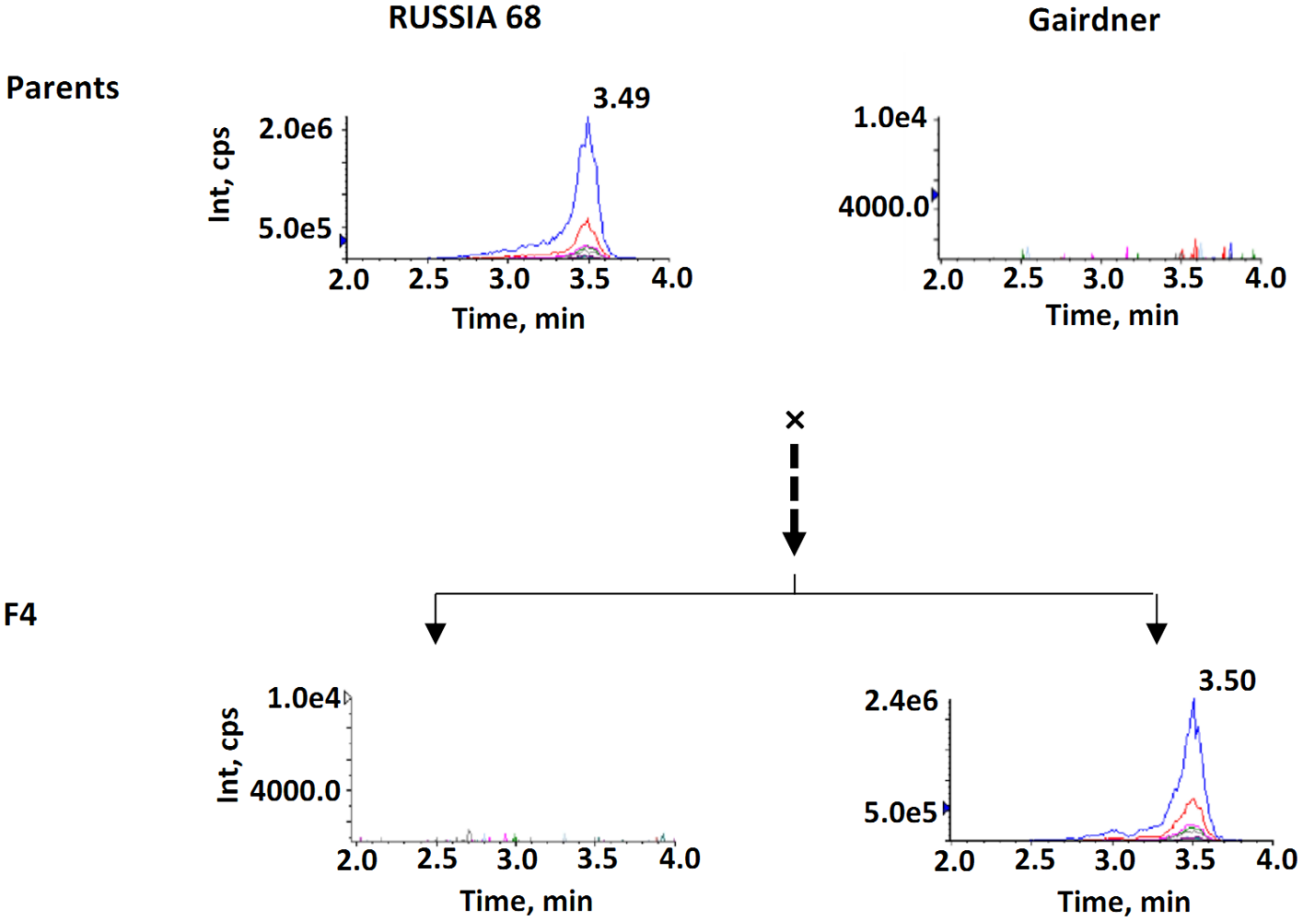
LC-MS profiles of peonidin-3-glucoside in the two parents and the two extremes in the F4 progeny of the mapping population. Int represents the signal intensity of the ions and cps the counts per second.

**Fig 3:**
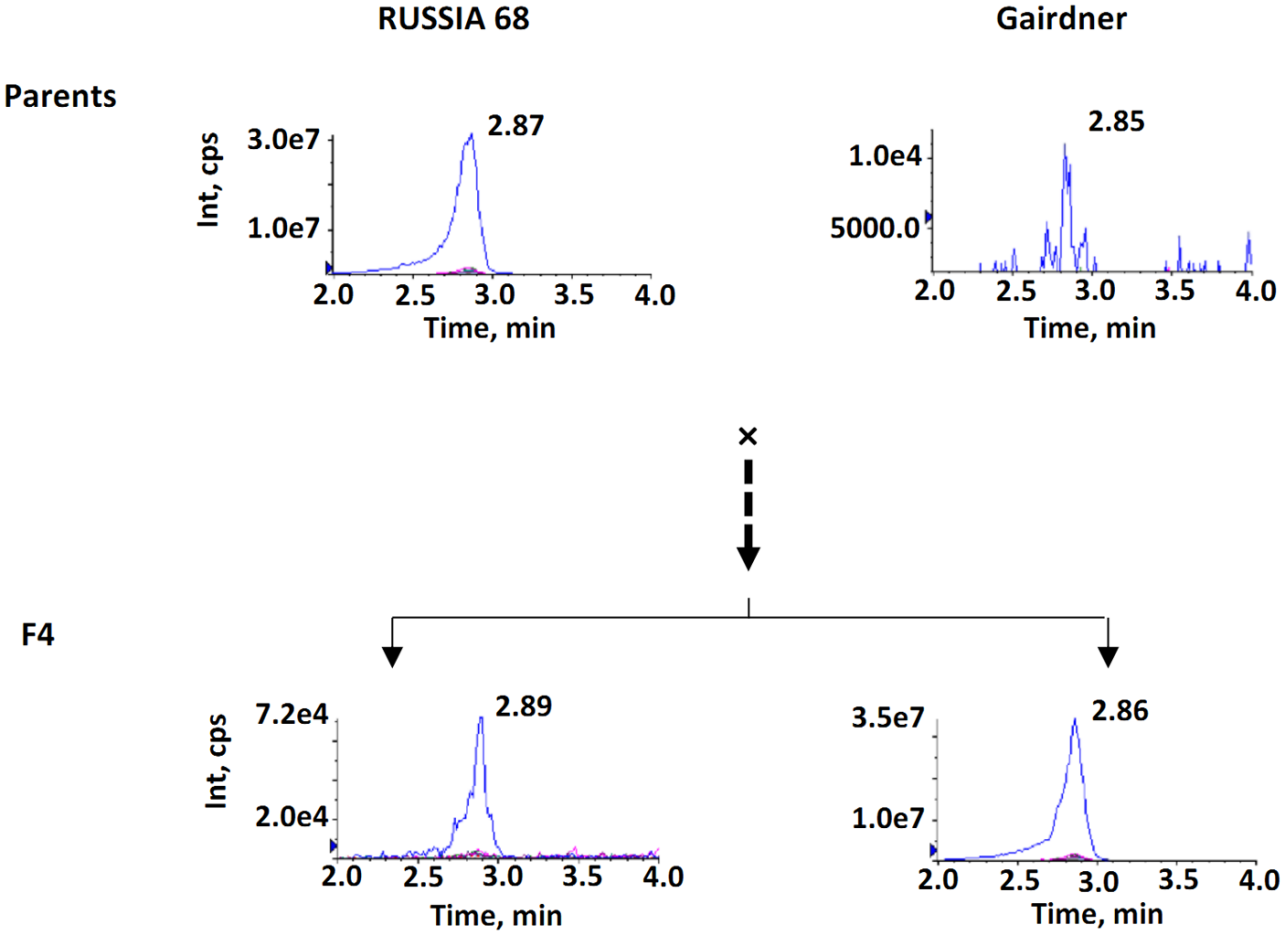
LC-MS profiles of cyanidin-3- glucoside in the two parents and the two extremes in the F4 progeny of the mapping population. Int represents the signal intensity of the ions and cps the counts per second.

Among the ten anthocyanin compounds assessed, seven (S1 Table) were detected in the grains of the purple pericarp genotype RUSSIA 68 and three were detected in the non-pigmented cultivar Gairdner. Two compounds, peonidin-3-glucoside (P3G) and cyanidin-3-glucoside (C3G), had strong signals in RUSSIA 68 and population derived from this genotype. P3G was detected in RUSSIA 68 but not in the female parent Gairdner (Fig 2). C3G was detected in both parents but it had a significantly (*p* < 0.05) higher level in the purple pericarp barley genotype (Fig 3). In POP1, the contents of P3G ranged from 0 to 5.07 μg per gram of dry weight and that of C3G ranged from 2.80 × 10^−3^ to 21.66 μg per gram of dry weight (Table 1).

**Table 1:** Concentration of two anthocyanins in extremes of POP1 ^a^^,^^b^.

Anthocyanin	RT (Min)	Purple grains	Non-colored grains
Cyanidin-3-glucoside (C3G)	3.49±0.02	20.581 ± 1.080	0.003 ± 0.001
Peonidin-3-glucoside (P3G)	2.87±0.02	5.310 ± 0.244	- ^c^

^a^ Concentrations are showed as micrograms per gram of dry weight.

^b^ Data are means ±SD.

^c^ “-” indicate the undetected anthocyanin.

### Identification of QTL conferring anthocyanin production

**Fig 4:**
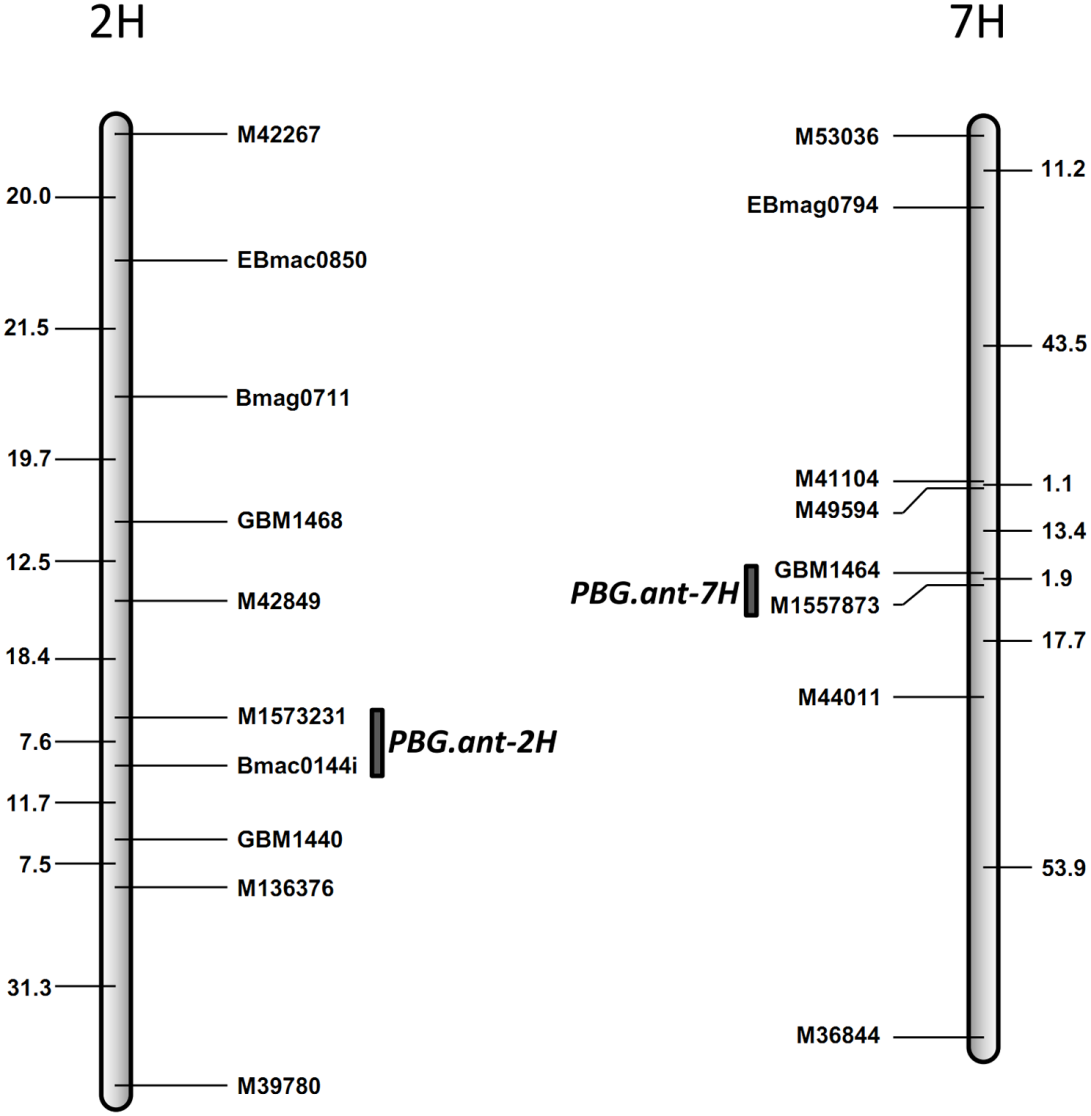
QTL conferring anthocyanin production detected on chromosome 2H and 7H in the mapping population. Marker positions are shown to the right of the linkage map and distances in centiMorgan (cM) between loci are shown to the left.

A total of 213 markers, including 145 SSR and 68 InDels, were selected based on their locations in previous constructed linkage maps. Among them, 51 were polymorphic between the parents of POP1 and they were used to genotype the whole mapping population. Based on the ‘single QTL’ model, a putative QTL was detected controlling both P3G and C3G. This QTL was derived from the purple pericarp barley parent Russian68 and located on the long arm of chromosome 2H (Fig 4, Table 2). It was designated as *PBG*.*ant-2H* following convention, where ‘PBG’ stands for ‘purple pericarp barley grain’ and ‘ant’ for ‘anthocyanin’. Markers flanking *PBG*.*ant-2H* were M1573231 and BMac0144i. This locus explained up to 57.1% of the P3G variance with a LOD score of 19.56 and up to 35.5% of the C3G variance with a LOD score of 9.97.

**Table 2:** QTL for anthocyanin production identified in the mapping population RUSSIA68/Gairdner (POP1).

Anthocyanin	QTL	Flanking markers	LOD	R^2^
Peonidin-3-glucoside	*PBG*.*ant-2H*	M1573231 & BMac0144i	19.56	57.1
	*PBG*.*ant-7H*	M1557873 & M44011	5.27	31.5
Cyanidin-3-glucoside	*PBG*.*ant-2H*	M1573231 & BMac0144i	9.97	35.5
	*PBG*.*ant-7H*	M1557873 & M44011	4.63	27.2

Further analysis was then conducted based on the model of ‘multiple QTL with interaction’ with the *PBG*.*ant-2H* locus as a main-effect QTL. When lines without any allele from the purple pericarp parent RUSSIA68 at the *PBG*.*ant-2H* locus were excluded from the mapping population, a second QTL derived from the purple pericarp parent was detected on the short arm of chromosome 7H (Fig 4, Table 2). This QTL, designated as *PBG*.*ant-7H*, explained up to 31.5% of the P3G variance with a LOD value of 5.27, and 27.2% of the C3G variance with a LOD value of 4.63.

**Fig 5:**
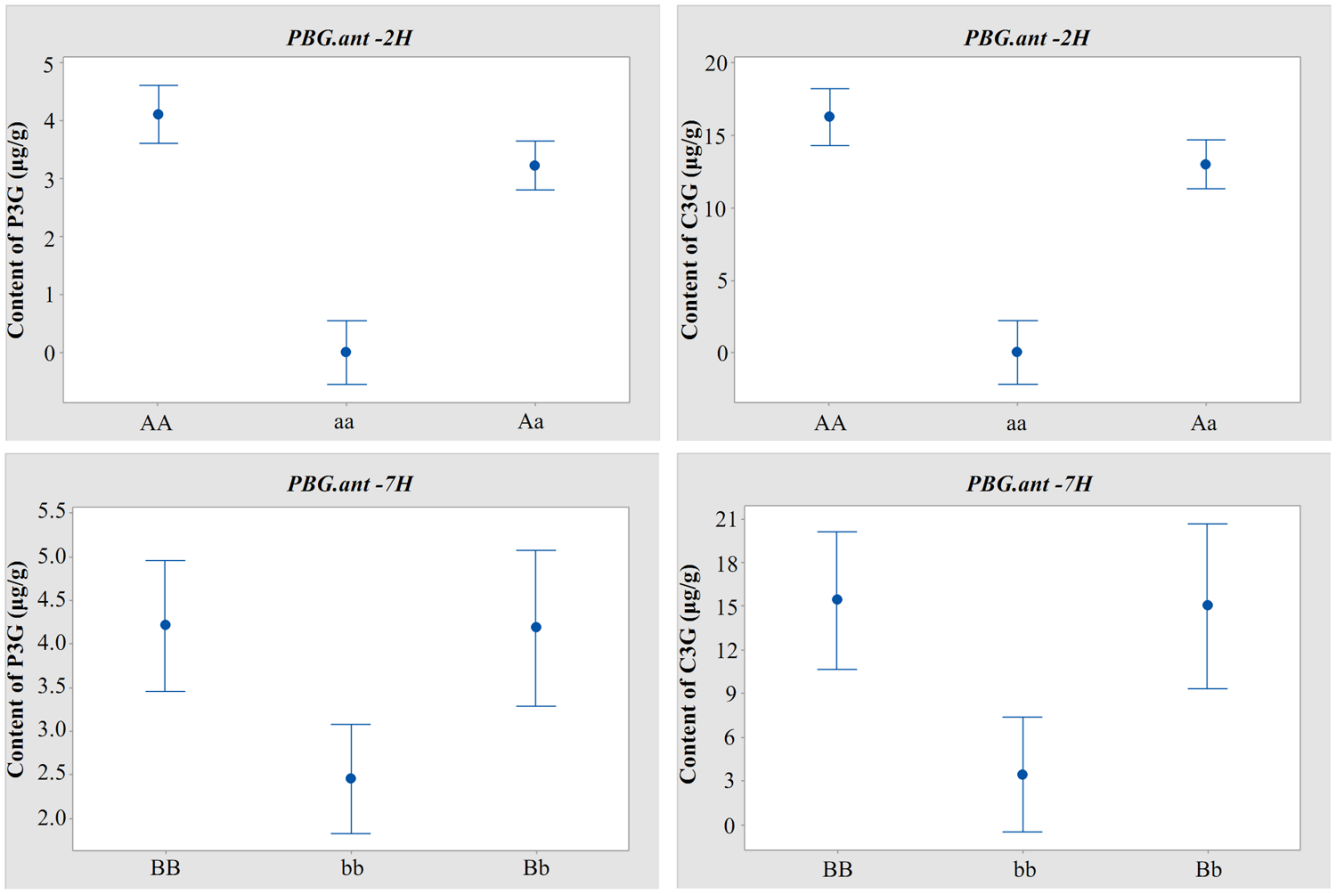
Effects of QTL for anthocyanin production determined by ANOVA (*p* < 0.05). ‘A’ and ‘a’ represent the presence or absence of *PBG*.*ant-2H* allele from the purple pericarp parent RUSSIA68 and the non-pigmented parent Gairdner, respectively; ‘B’ and ‘b’ represent the presence or absence of *PBG*.*ant-7H* allele from the purple pericarp parent RUSSIA68 and the non-pigmented parent Gairdner, respectively.

The effects of these QTL for either of the anthocyanin compounds in the mapping population were also assessed using analysis of variance (ANOVA). This analysis further demonstrated the presence of epistatic relationship between the two QTL. Irrespective of the status of the *PBG*.*ant-7H* locus, P3G was not detected from any of the lines without either of the *PBG*.*ant-2H* alleles from the purple pericarp parent. When one or both of the *PBG*.*ant-2H* alleles from the purple pericarp genotype was present, *PBG*.*ant-7H* alleles from the pigmented parent significantly (*p* < 0.05) increased the content of both P3G and C3G (Fig 5).

### Validation of the effects of *PBG*.*ant-2H* and *PBG*.*ant-7H* in a different genetic background

Possible effects of *PBG*.*ant-2H* and *PBG*.*ant-7H* were further assessed in the validation population (POP2) using the markers most closely linked to these two loci (M1573231 and M1557873, respectively). Significant effects were again detected for both of these QTL in this population (Table 3). *PBG*.*ant-2H* explained 50.6% of the P3G and 44.4% of the C3G variance with LOD scores of 14.21 and 11.77 respectively. *PBG*.*ant-7H* explained 21.4% of the P3G and 23.8% of the C3G variance with LOD scores of 3.51 and 4.00, respectively.

**Table 3:** Effect of QTL for anthocyanin production detected in the validation population Baudin//RUSSIA68/Gairdner (POP2).

Anthocyanin	QTL	Marker	LOD	R^2^
Peonidin-3-glucoside	*PBG*.*ant-2H*	M1573231	14.21	50.6
	*PBG*.*ant-7H*	M1557873	3.51	21.4
Cyanidin-3-glucoside	*PBG*.*ant-2H*	M1573231	11.77	44.4
	*PBG*.*ant-7H*	M1557873	4.00	23.8

## Discussion

The genetic control of two major anthocyanin compounds in barley was investigated in this study. Of the ten anthocyanin standards assessed, two (P3G & C3G) account for a large proportion of the TAC in the purple pericarp barley genotype RUSSIA68 for which segregating populations suitable for QTL mapping were generated. Both anthocyanins were the interactive products of two loci, one located on chromosome arm 2HL (*PBG*.*ant-2H*) and the other on 7HS (*PBG*.*ant-7H*). However, the two different anthocyanins seem to be controlled by different interactions between the two loci. The effect of the 7HS locus was hardly detectable without removing the effect of the 2HL locus. At least one copy of the 2HL alleles from the purple pericarp parent was required for the production of P3G. This does not seem to be the case for the production of C3G which was produced in any allele combinations between both of the loci. When one or both of the *PBG*.*ant-2H* alleles from the purple pericarp genotype was present, *PBG*.*ant-7H* alleles from the pigmented parent significantly (*p* < 0.05) increased the content of both P3G and C3G. It also became clear during the generation of the mapping and the validation populations used in this study that anthocyanin synthesis has strong maternal effect which was further confirmed from 4 additional crosses involving two different purple pericarp genotypes.

This is the first report on the genetics of individual anthocyanins in barley. However, investigating the genetic control of anthocyanins in pigmented barley has a long history. Based on segregation ratios of grain colour, early studies observed that pigmented grains could be controlled by a single locus or two loci [43, 44]. Chromosome arm 2HL was found containing genes controlling purple veined lemma or red lemma and pericarp [45]. A recent study also detected a purple grain locus mapped on 2HL [29]. It is not clear if the 2HL locus detected in this study was the same as the one reported in earlier studies. However, the fact that gene(s) conferring anthocyanin synthesis was detected on the same chromosome arm based on either colour segregation or the measurement of individual anthocyanin compounds indicating the significance of this chromosome arm in such investigations. In comparison, the effect of the 7HS locus detected in this study was novel but much weaker. Its effect was difficult to detect for both of the anthocyanin compounds investigated in this study without removing the effect of the 2HL locus. To our knowledge, the 7HS locus is novel. No loci conferring anthocyanin synthesis have been reported on this chromosome arm although a locus on the opposite arm of this chromosome was found responsible for the blue aleurone colour [28].

Nevertheless, several loci conferring anthocyanins have been reported. It is not unanticipated that additional loci will be discovered in future studies. Results on P3G and C3G from this study also showed that both the mode of gene action and the effectiveness can vary between loci. These results, coupled with previous findings that grains with similar colour can have highly different anthocyanin compositions [26] and that different anthocyanin compounds have different antioxidant activities [24, 34] thus different breeding and processing values, all point to the importance of targeting individual anthocyanin compounds in both genetics and breeding.

## Supporting information

S1 Table Anthocyanin compounds assessed in this study. (DOC)
