# Supplementary material for: Inheritance analysis and mapping of quantitative trait loci (QTL) controlling individual anthocyanin compounds in purple barley (Hordeum vulgare L.) grains
Source: PLoS One. 2017 Aug 23;12(8):e0183704. doi: 10.1371/journal.pone.0183704 (PMC5568277; doi:10.1371/journal.pone.0183704)
Supplement: S1 Table — (DOC) [file pone.0183704.s001.doc]

| **S1 Table. Anthocyanin compounds assessed in this study ^a,b^** | | | |
| --- | --- | --- | --- |
| **Anthocyanin** | **RUSSIA68** | **Gairdner** | **PubChem CID** |
| Cyanidin-3-glucoside (C3G) | 22.805±1.981 | 0.003±0.001 | 92131208 |
| Cyanidin-3-rutinoside | 0.012±0.002 | 0.002± 0.001 | 441674 |
| Delphinidin | - | - | 128853 |
| Delphinidin-3-glucoside | 0.141±0.017 | 0.017±0.002 | 443650 |
| [Malvidin](https://pubchem.ncbi.nlm.nih.gov/compound/69512) | - | - | 69512 |
| Malvidin-3-glucoside | 0.821±0.084 | - | 443652 |
| Pelargonin | 0.451±0.080 | - | 441772 |
| Peonidin | - | - | 441773 |
| Peonidin-3-glucoside (P3G) | 5.950±0.599 | - | 443654 |
| Petunidin-3-glucoside | 0.011±0.002 | - | 443651 |

^a^ Concentrations are showed as micrograms per gram of dry weight.

^b^ Data are means ±SD.

^c^ “-” indicate the undetected anthocyanin.
